# Biodiversity and Ecosystem Function Under Global Change: An Editorial for the Special Issue

**DOI:** 10.3390/biology14111503

**Published:** 2025-10-27

**Authors:** Shucun Sun, Panayiotis G. Dimitrakopoulos

**Affiliations:** 1 Department of Biology, School of Life Sciences, Nanjing University, Nanjing 210093, China; 2 Biodiversity Conservation Laboratory, Department of Environment, University of the Aegean, 81132 Mytilene, Greece

Climate change, land use changes, pollution (e.g., nitrogen deposition), and biological invasions are features of global environmental change, affecting biodiversity and ecosystem functioning worldwide [1]. In recent decades, many studies have been conducted to investigate how biodiversity loss affects ecosystem functioning under mean or variable environmental conditions [2,3,4,5]. However, the biological and ecological mechanisms through which global changes affect biodiversity and ecosystem functioning remain incompletely understood. This Special Issue, through its ten articles that cover a wide range of ecosystems—from alpine meadows and temperate steppes to semi-arid grasslands and polar marine environments— aims to address this gap by offering new knowledge about the mechanisms linking global change drivers to ecosystem structure and function.

Several research studies focus on understanding climate and nutrient interactions on carbon sequestration plant productivity, soil processes, and ecosystem dynamics in terrestrial ecosystems [6,7]. Bebaba et al. [8] showed that species richness and functional diversity of plants decreased with increasing nitrogen deposition rate and that nutrient enrichment affects the relationship between plant functional diversity and productivity but not that with taxonomic or phylogenetic diversity. Lou et al. [9] revealed that soil nitrogen supply levels affect variability in seed production and enhance reproductive success across years in a perennial herb in the eastern Tibetan plateau. Feng et al. [10] showed that long-term increases in daytime temperatures, more so than increases in nighttime temperatures, cause changes in the composition of soil microbial communities, reducing the ratio of fungi to bacteria, which enhances soil respiration in semi-arid grasslands.

Species interactions and ecological responses under changing environmental conditions is an important issue shaping the response of biological communities to global change [11]. An and Sun [12] found a clear impact of introduced Western honeybees on native bee species, especially rare species that present greater niche overlaps defined based on their food sources, underscoring that the development of beekeeping practices in natural ecosystems must consider the diversity and rarity of wild pollinators and the availability of food resources to them. Sun et al. [13] noted the role of rainfall timing but not rainfall intensity in the structure of dung beetle communities and the rate of dung decomposition in an alpine meadow in Tibet, explaining how changing precipitation patterns shape ecosystem function. Futai and Ishiguro [14] demonstrate that sexually mature *Monochamus* beetles are key drivers of pine wilt disease spread by transmitting nematodes to nearby healthy trees, leading to the formation of asymptomatic carriers that sustain and expand infections in pine forests.

On broader biogeographical scales, Abedin et al. [15] modeled the future loss and fragmentation of the habitat of the vulnerable Red Goral (*Naemorhedus baileyi*) due to climate change in the temperate mountains of South Asia, highlighting the need for effective conservation strategies. Focusing on Antarctic marine ectotherms, Morley et al. [16] investigated their thermal limits and physiological plasticity, providing critical information on the mechanisms of species-specific thermal tolerance in polar ecosystems. Bei et al. [17] present an example of adaptation of *Elaeagnus angustifolia* to arid environments, which has specialized umbrella-shaped trichomes on its leaves that allow it to collect water from the atmosphere while reflecting solar radiation, reducing water loss.

Finally, Pang et al. [18] present a bibliometric analysis of global research efforts (2002–2023) on carbon sources and sinks in farmland ecosystems, identifying emerging trends and research gaps in carbon management under global change.

Taken together, the studies presented in this Special Issue highlight the complex interconnections between biodiversity, ecosystem functioning, and environmental variability. Further research is needed to clarify how global drivers alter species interactions and ecosystem processes to guide effective conservation and policy responses to the alarming environmental changes influencing species and ecosystems worldwide.
